# Dissection of key factors correlating with H5N1 avian influenza virus driven inflammatory lung injury of chicken identified by single-cell analysis

**DOI:** 10.1371/journal.ppat.1011685

**Published:** 2023-10-11

**Authors:** Manman Dai, Sufang Zhu, Zhihao An, Bowen You, Ziwei Li, Yongxiu Yao, Venugopal Nair, Ming Liao

**Affiliations:** 1 National and Regional Joint Engineering Laboratory for Medicament of Zoonosis Prevention and Control, Guangdong Provincial Key Laboratory of Zoonosis Prevention and Control, College of Veterinary Medicine, South China Agricultural University, Guangzhou, China; 2 The Pirbright Institute and UK-China Centre of Excellence for Research on Avian Diseases, Pirbright, Ash Road, Guildford, Surrey, United Kingdom; University of Oxford, Oxford, United Kingdom; 3 Institute of Animal Health, Guangdong Academy of Agricultural Sciences, Guangzhou, China; The Peter Doherty Institute and Melbourne University, AUSTRALIA

## Abstract

Chicken lung is an important target organ of avian influenza virus (AIV) infection, and different pathogenic virus strains lead to opposite prognosis. Using a single-cell RNA sequencing (scRNA-seq) assay, we systematically and sequentially analyzed the transcriptome of 16 cell types (19 clusters) in the lung tissue of chickens infected with H5N1 highly pathogenic avian influenza virus (HPAIV) and H9N2 low pathogenic avian influenza virus (LPAIV), respectively. Notably, we developed a valuable catalog of marker genes for these cell types. Compared to H9N2 AIV infection, H5N1 AIV infection induced extensive virus replication and the immune reaction across most cell types simultaneously. More importantly, we propose that infiltrating inflammatory macrophages (clusters 0, 1, and 14) with massive viral replication, pro-inflammatory cytokines (IFN-β, IL1β, IL6 and IL8), and emerging interaction of various cell populations through CCL4, CCL19 and CXCL13, potentially contributed to the H5N1 AIV driven inflammatory lung injury. Our data revealed complex but distinct immune response landscapes in the lung tissue of chickens after H5N1 and H9N2 AIV infection, and deciphered the potential mechanisms underlying AIV-driven inflammatory reactions in chicken. Furthermore, this article provides a rich database for the molecular basis of different cell-type responses to AIV infection.

## Author summary

The low-pathogenicity avian influenza virus (LPAIV), H9N2, and highly pathogenic avian influenza virus (HPAIV), H5N1 are the main epidemic subtypes, resulting in great economic losses on poultry and potentially threat to human. Thus, in-depth exploration of AIV pathogenesis in chickens is necessary for developing efficient control methods. In our study, using a single-cell RNA sequencing analysis of 16 cell types in the chicken lung, we revealed complicated and distinct immune response landscapes after H5N1 and H9N2 AIV infection, and identified key factors contributing to H5N1 AIV driven inflammatory lung injury. Therefore, our study potentially provides new targets and direction for AIV control.

## Introduction

The low-pathogenic avian influenza virus (LPAIV), H9N2, and highly pathogenic avian influenza virus (HPAIV), H5N1 are the main epidemic subtypes in the ongoing virus circulation among Chinese poultry causing major economic losses in spite of the long-term vaccination programs [1–3]. Notably, the ongoing 2021–2022 wave of avian influenza H5N1 in Asia, Africa and Europe is unprecedented in its rapid spread and extremely high frequency of outbreaks in poultry [4]. More seriously, H5N1 virus can be transmitted from chickens to humans. Also, H9N2 virus can serve as the gene donor for H5N1, H7N9 and H10N8 viruses infecting humans [1–3,5–7]. Therefore, successful control of H5N1 HPAIV and H9N2 LPAIV in chickens is vital for the eradication of diseases and preventing infections in humans. Hence, it warrants deeper understanding of the determinant factors of avian influenza virus (AIV) infection and pathogenesis in chickens for developing efficient control methods.

AIV infections of the chicken mainly occur via the respiratory route, and the lung is the important target organ. In lung tissue, AIV infection induces both antiviral and inflammatory factors which play crucial roles in host protection and immunopathogenesis [8,9]. Infection of chickens with the H9N2 LPAIV usually results in mild clinical signs whereas H5N1 HPAIV induces death within 36–48 hours, which is related to heightened inflammatory responses in the latter [10]. However, the specific immune cell types and inflammatory factors contributing to the immune injury of chicken lung are poorly understood. Besides, efficient replication of HPAIV compared to LPAIV has been correlated with tissue damage in the lung [11,12]. But the extent and nature of HPAIV and LPAIV infection in different cell types of chicken lung has not been elucidated. In this study, we want to explore the spectrum of cell infection types, and the immune reaction profiles of chicken lung at the peak of H5N1 HPAIV and H9N2 LPAIV infections.

Single-cell RNA sequencing (scRNA-seq) has been widely applied to identify the involvement of different cell types and to investigate the immune responses under conditions of virus infection [13–15]. It is also a powerful tool for defining viral target cells via analyzing the viral mRNAs and host signature genes in a single cell [13,16–18]. In particular, scRNA-seq can precisely be used to examine the patterns of cytokine release in each immune cell and intercommunication with the ligand and receptor interaction map at the single cell level [13,19,20]. To our knowledge, this is the first study on the application of scRNA-seq technology to examine the responses of the major immune cell types in the lung tissue of chickens infected with H5N1 and H9N2 AIV.

In order to explore more comprehensive and refined immune cell responses to H5N1 and H9N2 AIV infection, we sorted the major immune cell types, including MHC Class II antigen presenting cells and CD3 positive T cells from chicken lung mononuclear cells for performing scRNA-seq analysis. In this study, we analyzed 16,642 immune cells in the lung tissue isolated from chickens after AIV infection with different pathogenic strains at various time points. Our results revealed complex but distinct immune response landscapes in the lung tissue of chickens after H5N1 and H9N2 AIV infection. Our study has developed a valuable catalog of marker genes for identifying 16 cell types in the lung tissue of chicken via scRNA-seq. We also provided (for the first time) the key immune cell types and pro-inflammatory factors that contribute to H5N1 AIV-driven inflammatory injury in chicken lung tissue.

## Materials and methods

### Ethics statement

All animal experiments were carried out in ABSL-3 facilities in compliance with approved protocol (CNAS BL0011) by the biosafety committee of South China Agriculture University (Guangzhou, China). All animal procedures were performed according to the regulations and guidelines established by this committee and international standards for animal welfare.

### Virus and experimental animal infection

Low pathogenic avian influenza H9N2 subtype HN strain (A/Chicken/Hunan/HN/2015), and high pathogenic avian influenza H5N1 subtype DK383 strain (A/Duck/Guangdong/383/2008) were isolated and identified by our research team [21,22]. Nine four-week-old, specific pathogen–free (SPF) White Leghorn chickens (Guangdong Da Hua Nong Animal Health Products Co., Ltd., Guangdong, China) were randomly assigned to three groups, namely, a H9N2-infected group, a H5N1-infected group and control group, each with 3 chickens. The H9N2-infected group was intranasally (i.n.) inoculated with HN strain (10^7^ EID50 in 0.2 mL). The H5N1-infected group was intranasally inoculated with DK383 strain (10^6^ EID50 in 0.2 mL). The control group was inoculated with 0.2 mL of phosphate buffered saline (PBS). Three chickens were dissected at 1-day post-inoculation (DPI) in the H5N1-infected group, 3 DPI in the H9N2-infected group, and 0 DPI in the control group, respectively. The virus titer in selected organs and virus shedding were detected as previously described [2], and the data are shown in S1 File.

### Immunohistochemistry (IHC) and Preparation of chicken lung mononuclear cell suspension

Infected or mock-infected chickens were humanely sacrificed. Their lungs were then perfused with RPMI-1640 containing 2% fetal bovine serum (FBS) (Invitrogen, Waltham, MA, USA) via the right ventricle. Half of the entire pool of the lung was fixed in 4% paraformaldehyde for 24 hours and embedded in paraffin. Tissue slides were deparaffinized with xylene, hydrated in a graded series of alcohol solutions to distilled water, and blocked for endogenous peroxidase in 3% hydrogen peroxide. Sections were then stained with mouse anti-Influenza A virus NP primary antibody (GeneTex, Alton, CA, USA), followed by staining with anti-mouse-HRP secondary antibody (Zhongshan Goldenbridge, Beijing, China) and visualizing via optical microscopy (Olympus, Tokyo, Japan). Then, 0.1g from the other half of the lung tissue was kept for virus titer detection, and the remaining half of the lung pool was dissected and dissociated into single-cell suspensions, using the Lung Dissociation Kit (Miltenyi Biotec, Bergisch Gladbach, Germany) in combination with a gentleMACS dissociator (Miltenyi Biotec) and enzymatic dissociation [23]. Following enzymatic incubation, cells were forced through a 70-μm mesh cell strainer. After that, lung mononuclear cells were enriched from lung suspension with the tissue mononuclear cell kit (Haoyang, Tianjin, China), as described previously [2,24]. Cell viability and counting was performed using Trypan Blue and hemocytometer (Sigma-Aldrich).

### Sample and library preparation for 10x scRNAseq

3×10^7^ lung cell pooled suspension was prepared from three chickens, with 1×10^7^ lung cell suspension for each treatment group. MHC Class II and CD3 positive cells were sorted out from each pulmonary cell pool after incubation with the FITC-conjugated MHC Class II and APC-conjugated CD3 antibodies (SouthernBiotech, Birmingham, AL) using Fluorescence-activated cell sorting machine (FACS Aria II, Becton Dickinson, New Jersey, USA). The gating strategy for each population is shown in S1 Fig. Equal number of MHC Class II and CD3 positive cells in each pulmonary cell pool were mixed together, followed by passing through a 40 μm cell strainer (Biosharp, China), and the cell viability was above 80%. Subsequently, the cell density was adjusted to 1 x 10^6^ cells/mL. High quality single cell suspension was subjected to encapsulation using a 10x Genomics v.3 kit (10x Genomics, USA). The library preparation and RNA-sequencing were completed by Gene Denovo (Guangzhou, China) as described previously [15]. An average of 32956 reads per cell in the H9N2 group, mean reads of 35911 per cell in the H5N1 group, and mean reads of 28419 per cell in the control group were obtained, respectively.

### FACS analysis of Macrophages in lung mononuclear cell suspension

The percentage of macrophages from lung single cell suspension was detected via flow cytometry (CytoFLEX, Beckman Coulter, Brea, CA, USA) with the FITC-conjugated mouse anti-chicken MHC Class II and PE-conjugated mouse anti-chicken KUL01 monoclonal antibodies (Southern Biotech, Birmingham, USA) [25]. Data were analyzed by the software of FlowJo V10 (TreestarInc, Ashland, OR, USA). Absolute number of macrophages was calculated by multiplying the percentage of macrophages with the total cell number of single cell suspension isolated from lung.

### Macrophage sorting and generation of macrophage reference transcriptional profile via SMART-Seq2 based scRNA-seq

Fluorescence-activated cell sorting machine (FACS Aria II, Becton Dickinson, New Jersey, USA) was used to sort a single cell into each well of a 96-well PCR plate containing 2.5μL of 10× Lysis Buffer (Vazyme# N711). For the isolation of macrophage, each pulmonary cell pool was stained with FITC-conjugated mouse anti-chicken MHC Class II and PE-conjugated mouse anti-chicken KUL01 monoclonal antibodies (Southern Biotech, Birmingham, USA). Macrophages were sorted following the manufacturer’s procedures. Herein, each sorted cell population was analyzed in four replicates, and 100 single cells were sorted in each replicate for subsequent SMART-Seq2 analysis. Four empty wells served as controls in each 96-well plate. Immediately after sorting, each plate was spun down to ensure immersion of cells into the lysis solution, snap-frozen on dry ice, and stored at −80°C until processing. Library construction and sequencing were completed by Gene Denovo (Guangzhou, China) as described previously [26]. Pearson’s correlation analysis was used to investigate the relationships between the SMART-Seq2 data of macrophages and lung clusters in scRNA-seq based on levels of gene expression.

### Quantitative Reverse Transcription PCR (qRT-PCR)

Total RNA was extracted from lung cell suspensions or sorted macrophages for qRT-PCR. qRT-PCR was performed on an ABI7500 Real-Time PCR system (Applied Biosystems, Waltham, MA) using Universal SYBR R Green Supermix Kit reagents (Biorad, CA, USA) according to the manufacturer’s specifications. Primers used for qRT-PCR are listed in S2 File. Data analysis were performed using the 2^-ΔΔ*C*t^ method [27].

### 10x scRNAseq sequencing data analysis

As described previously [15], the raw scRNA-seq data were aligned, filtered, and normalized using Cell Ranger (10x Genomics) software (Cell Ranger 3.1.0), and the cDNA reads were mapped to the chicken genome of GRCg6a [28]. Only reads that were confidently mapped to the transcriptome were used for Unique Molecular Identifier (UMI) counting. Cells with unusually high numbers of UMIs (≥8000) or mitochondrial gene percentage (≥20%) were filtered out. Cells with <500 or >4000 gene counts were also excluded. Seurat (v4.0.4) is a popular R package that was used to implement the graph-based clustering approaches [29,30]. T-distributed Stochastic Neighbor Embedding (t-SNE) [31] or Uniform Manifold Approximation and Projection (UMAP) [32] in Seurat were used to visualize and explore these datasets. Other data analyses including standardization, difference of gene expression, and marker gene screening were also achieved by Seurat.

### Cluster marker analysis and cell type annotation

Four strategies were used for cell-type annotation. Firstly, we identified cell types based on the top 5 expressed genes in each cluster and classical marker genes of chicken immune cells, e.g: CD4 T cells (*CD3D*, *IL7R* and *CD4*), B cells (*BCL11A*, *Bu-1* and *BLB2*), CD8 T cells (*CD3D*, *CD8A* and *GNLY*), Cytotoxic T cells (*CD3D*, *CD8A*, *GNLY*, *GranzymeA* and *IFNG*), and Dendritic Cells (DCs, *BLB2*, *CD80*, *CD86* and *XCR1*) [33,34]. Secondly, the identity of some cell clusters was manually annotated based on the specific expression of the marker genes that had orthologous genes as commonly known markers published in the CellMarker database (http://iocc.hrbmu.edu.cn/CellMarker/) [35], e.g: Th17 cells (*CD3D*, *RORA*, *CCR6* and *CCL20*), Macrophages (*IL1B*, *VCAN*, and BLB2), and Smooth muscle cells (*MYH11* and *TAGLN*). Next, identification of most of the remaining cell clusters was based on the specific expression of the marker genes reported in the literature, e.g: Epithelial cells (*BMX* and *EHF*) [36], type II alveolar epithelial cells (*SFTPA1*, *SFTPA2* and *SFTPC*) [37–39], Fibroblasts (*COL1A1*, *COL1A2* and *COL6A1*) [14], Vascular endothelial cells (*ADGRL4*, *VWF*, *PODXL* and *SELECTIN*) [40–42], M2 macrophages (*BLB2*, *RNASE6* and *VSIG4*) [43,44], and Regulatory T cells (Tregs, *CD3D*, *CD25*, *IL7R* and *TNFRSF1B*) [45,46]. Last, we combined the marker genes in the CellMarker database with reported distinctive genes in the literature for annotating the remaining clusters, e.g: Macrophage like cells (*BLB2*, *VCAN*, *IL1B*, *HSPH1* and *DNAJA4*) [47], and Th2 cells (*CD3D*, *MAF*, *CCR4*, *DRD4*, and *KK34*) [15]. The distribution of the characteristic gene expression in each cluster was then demonstrated using heat maps and bubble diagrams. The detailed marker gene information for 18 clusters and cell type annotation is shown in S3 and S4 Files.

### Viral Gene Analysis in Cell Clusters of H9N2-infected group, H5N1-infected group and control group

To determine the state of H9N2 AIV or H5N1 AIV infection in each cell type, the expression of viral genes and viral load were analyzed in the different cell types in the H9N2-infected group, H5N1-infected group and control group. Viral gene expression was analyzed using Cell Ranger, based on the viral sequence of H9N2 subtype HN strain (A/Chicken/Hunan/HN/2015) or H5N1 subtype DK383 strain (A/Duck/Guangdong/383/2008) used in this study. The ‘viral genome load’ of a cell in scRNA-seq analysis was based on the number of UMIs that mapped to the AIV eight segmented mRNAs and expressed as a percentage of total UMI content of a given cell. To reduce the false positive rate of infected cells, only the cells with highly expressed AIV genes (at least one transcript per gene per cell) were defined as highly infected cells. Therefore, the cells in each cell type susceptible to AIV infection could be divided into highly infected cells (I, total UMI counts of viral transcripts ≥ 8), potential or lowly infected cells (P, total UMI counts of viral transcripts ≥ 1), and undetected cells (N, UMI counts of viral transcripts = 0) [13].

### Differentially Expressed Genes (DEGs) Analysis in each cell type between H5N1 or H9N2-infected and uninfected groups

To explore the response of each cell type to AIV, we further analyzed the DEGs between the H5N1 or H9N2-infected group and the control group using Seurat software. A hurdle model in MAST (Model-based Analysis of Single-cell Transcriptomics) [48] was used to find DEGs for a group in each cell type. DEGs between the H5N1or H9N2-infected group and control groups were identified by the following criteria: 1) |log2FC|≥0.36; 2) p value_adj ≤ 0.05; and 3) percentage of cells where the gene was detected in a specific cluster ≥ 10%. Identified DEGs were subsequently subjected to GO enrichment analysis.

### Ligand-receptor intercellular communication network analysis

We used cellphone DB [49] to infer ligand-receptor pairs in chicken cells via orthologous gene analysis. A ligand/receptor with non-zero expression in more than 10% of cells in a particular cell population was deemed an “expressed” ligand/receptor. Then igraph in R was used to draw interaction maps as previously described [50]. In order to show the lost and/or new interaction after H5N1 or H9N2 AIV infection treatment, we calculated all the interactions for control and the treatment (H5N1 or H9N2 AIV) and then generated an interaction map from all interactions of control and the treatment. Then, we colored the edges as cyan (lost after treatment), purple (induced after treatment) and black (unaffected by treatment but may be differentially expressed).

### Statistical analysis

Data were analyzed using GraphPad Prism 8.0 software (GraphPad Software Inc., San Diego, CA, USA). The results were presented as mean ± SEM. The paired t-test and one-way ANOVA were used for statistical comparison. Data were considered significant at * *P* < 0.05, ** *P* < 0.01, *** *P* < 0.001.

## Results

### Identification of cell clusters in the lung tissues collected from H9N2 AIV infected, H5N1 AIV-infected and control chickens

**Fig 1:**
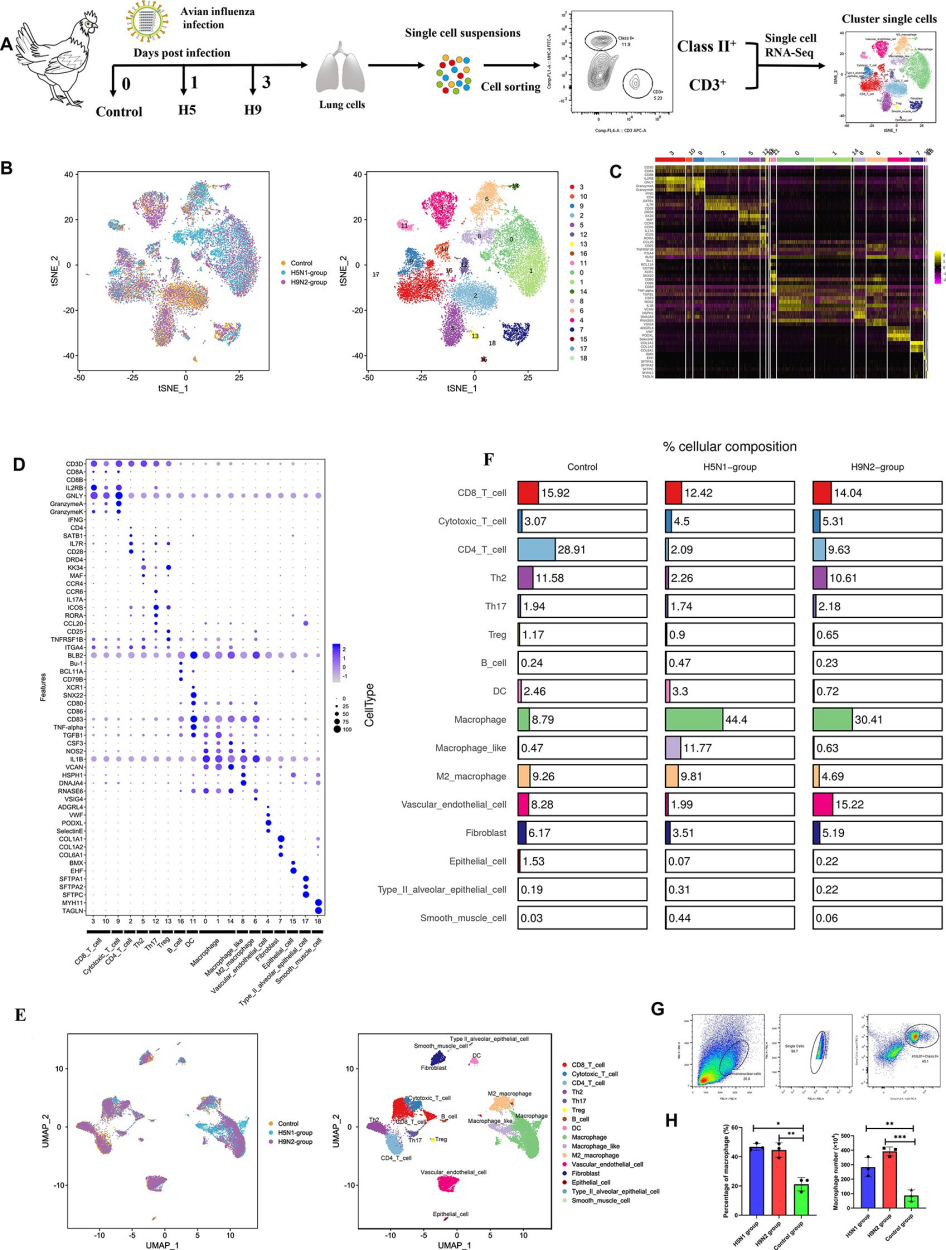
Single-cell profiling of cell populations in the lung collected from H9N2 AIV infected, H5N1 AIV infected and control chickens. (A) Overview of the study design. The lung cell suspension from three chickens were mixed together as the pulmonary cell pool for each treatment group. Equal number of MHC Class II and CD3 positive cells in each pulmonary cell pool were sorted out and mixed together as one sample for single-cell sequencing analysis. (B) t-Distributed Stochastic Neighbor Embedding (t-SNE) projection representing the 19 clusters of cells identified in the chicken pulmonary cell pools (unified set of control, H9N2 AIV and H5N1 AIV infection samples). (C) Heatmap showing the normalized expression (Z-score) of characteristic genes in each cluster. (D) Cell type annotation and dot plot representing characteristic genes (y-axis) in each cluster (x-axis). Dot size represents the proportion of cells in the cluster that express the gene; intensity indicates the mean expression level (Z-score) in the cells, relative to those from other clusters. (E) Uniform Manifold Approximation and Projection (UMAP) displaying all identified cell types. (F) Table displaying the total contribution of each cell type aggregated for the control, H5N1 group and H9N2 group samples in percentage of prepared cell suspension, respectively. (G) Gating of macrophages with the KUL01^+^ (PE) and MHC Class II^+^ (FITC) antibodies. (H) The percentage or number of macrophages from lung single cell suspensions in the H5N1 group, H9N2 group and Control group, respectively. The data were collected from three biological samples. Statistical analysis was performed using one-way ANOVA. *P < 0.05, **P < 0.01, ***P < 0.001.

To investigate the responses of major immune cell populations in the lung tissue isolated from chickens infected with different pathogenic AIV strains at the peak of viral infections, we collected pooled MHC Class II (antigen presentation cells) and CD3 (T cells)-positive cells derived from the lungs of low pathogenic H9N2 AIV-treated (3 DPI), highly pathogenic H5N1-treated (1 DPI), and PBS-treated (Control) chickens, for scRNA-seq transcriptional profiles using the 10× Genomics platforms (Fig 1A). Details on the statistical analysis of scRNA-seq data are summarized in S5 File. A total of 19,451cells (H9N2 AIV-infected: 6,936; H5N1 AIV-infected: 6,779; Control: 5,736) were profiled and 19 distinct clusters that could be visualized using t-SNE were obtained (Fig 1B). The heatmaps displayed the expression level of characteristic genes in each cluster (Fig 1C). Also, we manually annotated each cluster based on the expression of characteristic marker genes (as described in the materials and methods section on strategies for cell-type annotation) (Fig 1D and S3 and S4 Files). The result showed that there was 5 clusters of non-immune cell types: Vascular endothelial cell (Cluster 4), Fibroblast (Cluster 7), Epithelial cell (Cluster 15), type II alveolar epithelial cell (Cluster 17), and Smooth muscle cell (Cluster 18), and 11 specialized immune cell types: CD8^+^ T cell (Cluster 3 and Cluster 10), Cytotoxic T cell (Cluster 9), CD4^+^T cell (Cluster 2), Th2 cell (Cluster 5), Th17 cell (Cluster 12), Treg cell (Cluster 13), B cell (Cluster 16), DC (Cluster 11), Macrophage (Cluster 0, Cluster 1 and Cluster 14), Macrophage like cell (Cluster 8), and M2 Macrophage (Cluster 6) (Fig 1D and S3 and S4 Files, cell type annotation methods). Thus, the atlas of 16 major cell subsets in the lung was also displayed with UMAP (Fig 1E).

To explore the effect of AIV infection on the composition of cell subsets in the lungs, we analyzed the proportion of each cell type in the lung from H9N2 AIV infected, H5N1 AIV infected and control chickens. We found that the proportion of macrophages showed a huge increase in the lungs from H9N2 AIV infected or H5N1 AIV infected chickens when compared to the control (Fig 1F). Moreover, we found that the changes in the macrophages from single cell suspensions of lungs during H9N2 AIV or H5N1 AIV infections determined by FACS analysis were similar with that in scRNA-seq data (Fig 1G and 1H), which further confirmed the scRNA-seq data and indicated the importance of macrophages in AIV infection and pathogenesis.

### The global infection signature of cell types in the lung after H9N2 or H5N1 AIV infection

**Fig 2:**
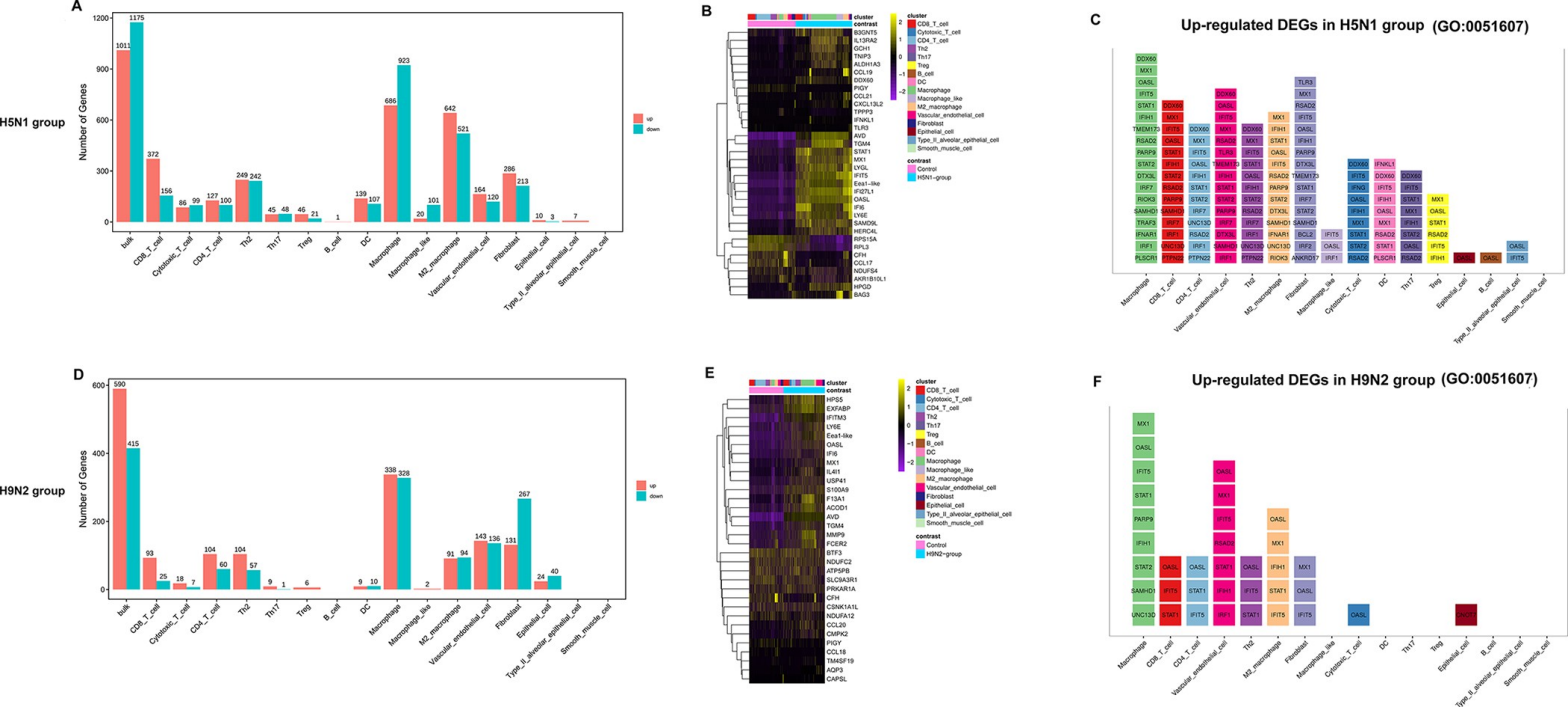
Global infection signature of cell types in the lung after H9N2 or H5N1 AIV infection. Histogram showing the number of up-regulated (red) and down-regulated DEGs (green) in H5N1-infected cells (A) or H9N2-infected cells (D) compared to control cells within each cell type. Heatmap showing the normalized expression (Z-score) of the top DEGs in H5N1-infected cells (B) or H9N2-infected cells (E) compared to control cells within each cell type. Histogram showing the significantly up-regulated DEGs (log2 fold change≥0.36, and p value_ adj≤0.05) in each cell type enriched in the gene ontology term of “defense response to virus” (GO: 0051607). The ranking of genes from top to bottom is based on the mean expression level in H5N1-infected cells (C) or H9N2-infected cells (F) compared to control cells within each cell type.

To characterize the host responses to H9N2 or H5N1 AIV infection, we analyzed the differential expressed genes between cell populations of the H9N2 or H5N1 AIV-treated and control samples using Seurat package (v4.0.4). Massive changes of the transcriptional landscape were found between the normal lung tissue and tissue from chickens challenged with H9N2 AIV or H5N1 AIV. Compared to the control sample, it was found that most up-regulated host genes were in macrophages and M2 macrophages of H5N1 group, and macrophages of H9N2 group (Fig 2A and 2D, and S6 File). And the number of DEGs in most cell populations of H5N1 group was obviously higher than that in the H9N2 group (Fig 2A and 2D), which implied that H5N1 AIV infection induced more robust host responses than H9N2 AIV infection. Then, we curated a global infection signature of top DEGs in H5N1 group and H9N2 group, with varying degree of specificity to a certain cell type (Fig 2B and 2E). We further verified 10 selected up-regulated genes across all cells in the lung after AIV infection by qPCR, which further confirmed the scRNA-seq data (S2 Fig). We also analyzed the expression of genes involved in the “defense response to virus” (GO: 0051607) within various cell types of H5N1 group and H9N2 group. Antiviral gene expression profiles for each cell type after AIV infection are displayed in Fig 2C and 2F. Macrophages generated the most antiviral factors, and H5N1 AIV infection induced a stronger antiviral immune response than H9N2 AIV infection in lung (Fig 2C and 2F, and S7 File).

### Viral load detection in various cell types of H5N1 group, H9N2 group and Control group

**Fig 3:**
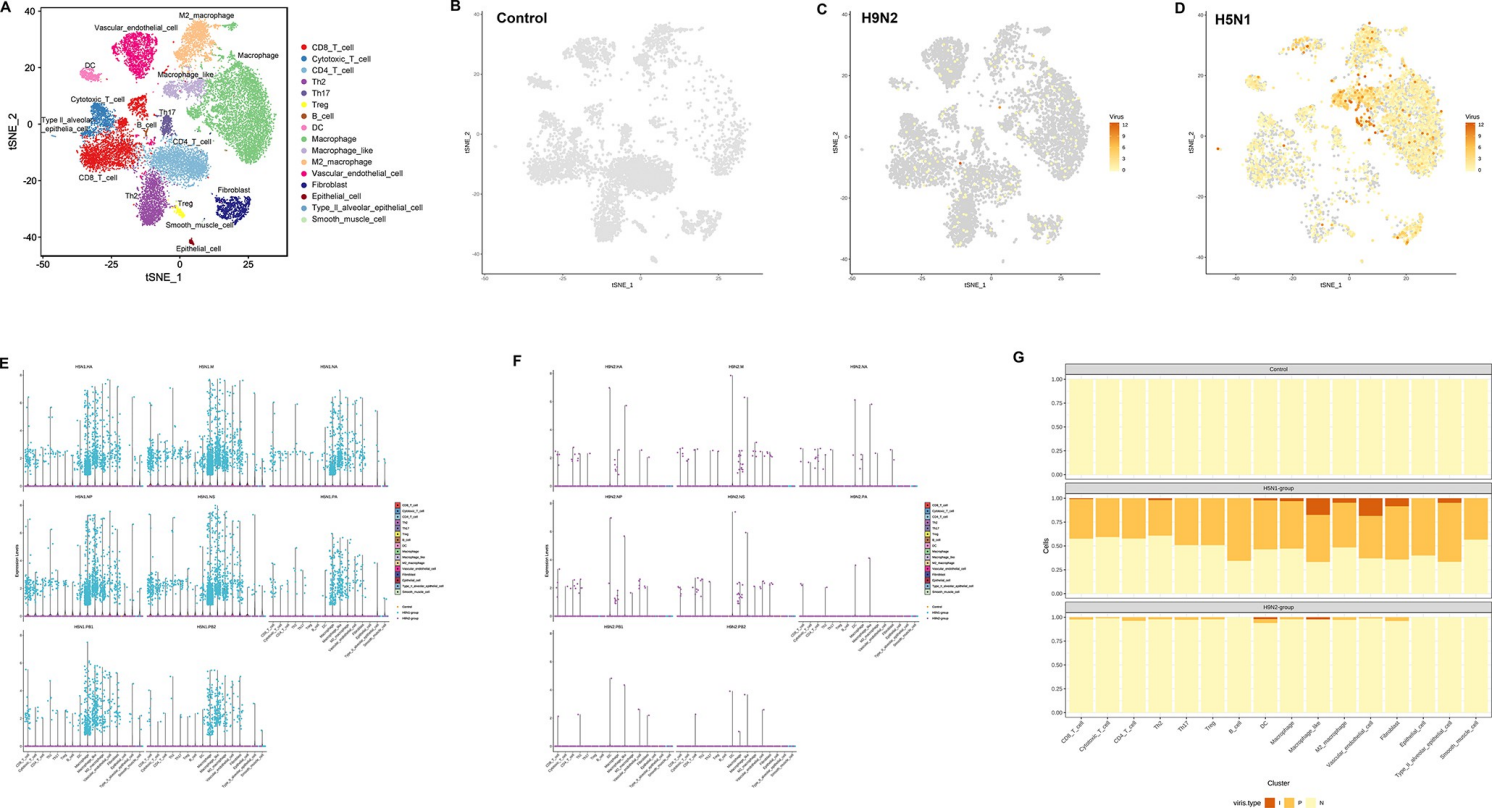
Viral load detection within various cell populations of H5N1 group, H9N2 group and Control group. (A) t-Distributed Stochastic Neighbor Embedding (t-SNE) projection representing 16 cell types. t-SNE displaying the normalized expression (Z-score) of viral genes in control cells (B), H9N2-infected cells (C), and H5N1-infected cells (D). Violin plots showing the expression levels of H5N1 AIV genes (E) or H9N2 AIV genes (F) in various cell types. (G) The cells in various cell types susceptible to AIV infection were divided into highly infected cells (represented by I, total UMI counts of viral transcripts ≥ 8), potential or lowly infected cells (represented by P, total UMI counts of viral transcripts ≥ 1), and undetected cells (represented by N, UMI counts of viral transcripts = 0). The percentages of highly infected cells (brown), potential or lowly infected cells (yellow), and undetected cells (light yellow) were shown in y axis.

Since the viral mRNA is poly-adenylated, scRNA-seq can capture both viral and host mRNAs within each individual cell. Therefore, host cells infected with AIV were quantitatively identified by tracking the intracellular AIV-segmented mRNAs at single-cell resolution. In Fig 3A–3D, the abundance of viral gene expression in individual cells is illustrated. Viral genes could be detected in a large number of cells of the H5N1-infected sample, but not H9N2-infected sample, illustrating widespread and efficient replication of H5N1 AIV in the lung (Fig 3A–3D). To better understand the infected cells in lung, the UMI counts of AIV genes were sought in single cell transcriptional data (S8 File). The violin plots showed that eight viral genes were all highly expressed in macrophage, macrophage-like and M2 macrophage populations of H5N1 group (Fig 3E and S8 File). Conversely, few viral genes were expressed in cells of H9N2 group (Fig 3F and S8 File). According to the expression counts of AIV transcripts, the cells in the clusters susceptible to AIV infection could be divided into highly infected cells (I, total UMI counts of viral transcripts ≥8), potentially or lowly infected cells (P, total UMI counts of viral transcripts ≥1), and undetected cells (N, UMI counts of viral transcripts = 0). It was observed that nine of the infected cell populations (CD8 T cell, Th2, DC, Macrophage, Macrophage like, M2 Macrophage, Vascular endothelial cell, Fibroblast and type II alveolar epithelial cell) of H5N1 group carried a high viral load (Fig 3G). On the other hand, only two of the infected cell populations (DC and Macrophage like) of H9N2 group carried a high viral load (Fig 3G). Additionally, the proportion of infected cells in the H5N1 group was much higher than that in the H9N2 group (Fig 3G). Immunohistochemistry (IHC) results showed abundant viral protein expression in the lung in the H5N1 group, compared to the H9N2 group (S3 Fig, IHC). Moreover, hematoxylin and eosin (HE) staining results indicated that there were significant lesions in the lung of the H5N1 group, including congestion and inflammatory cell infiltration, but no obvious lesions were found in the lung of H9N2 group (S3 Fig, HE). These results reminded that massive viral replication of H5N1 AIV in the chicken lung induced inflammatory damage.

### The landscape of inflammatory response within each cell type in the lung after H9N2 or H5N1 AIV infection

**Fig 4:**
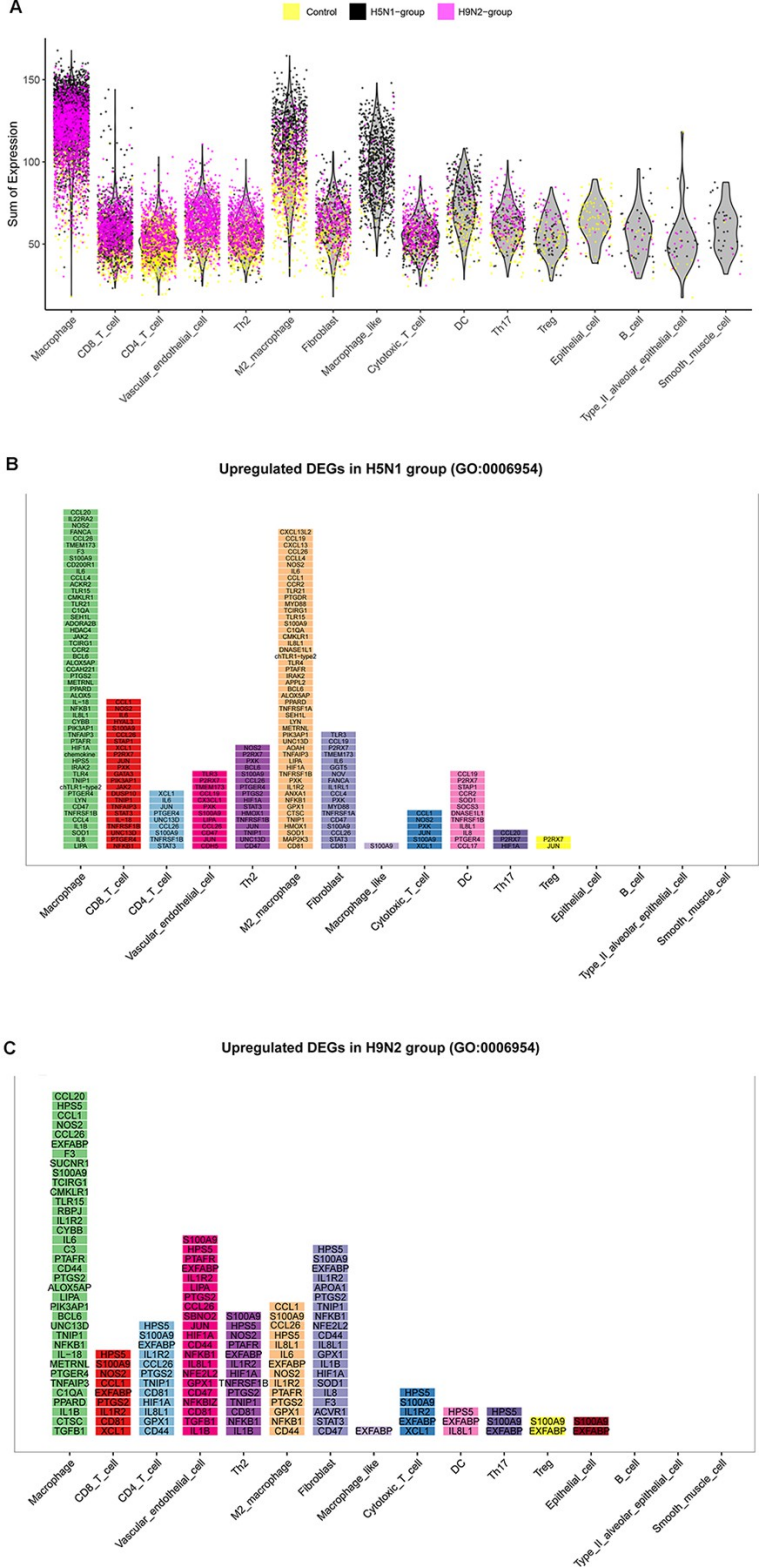
The landscape of inflammatory response within each cell type in the lung after H5N1 or H9N2 AIV infection. (A) The sum UMI counts expression of host 301 genes related to inflammatory response in different cell types (X-axis) (GO: 0006954). The dots indicate the cells from different groups, colored according to the samples. (B) Up-regulated genes (log2 fold change ≥0.36) in each cell type of H5N1 group enriched in the gene ontology term of “inflammatory response”. The ranking of genes from top to bottom is based on the mean expression level in each cell type. (C) Up-regulated genes (log2 fold change ≥0.36) in each cell type of H9N2 group enriched in the gene ontology term of “inflammatory response”. The ranking of genes from top to bottom is based on the mean expression level in each cell type.

AIV infection initiates in the respiratory tract and spreads in the lung, which triggers widespread pulmonary immune responses. Our foregoing results indicated that highly pathogenic H5N1 AIV, instead of low pathogenic H9N2 AIV infection, induced pulmonary inflammatory damage and a strong antiviral immune response with major viral replication. Therefore, we further analyzed the expression of genes involved in the inflammatory response after AIV infection. There are 301 genes related to the GO term of “inflammatory response” (GO: 0006954). The inflammatory response was firstly analyzed within various cells. And we found that macrophage, M2 macrophage and macrophage-like populations generated abundant inflammatory factors (Fig 4A). Then, up-regulated genes related to inflammatory response in each cell type of H5N1 group or H9N2 group are displayed (Fig 4B and 4C, and S9 File). The ranking of gene from top to bottom is based on the mean expression level in each cell type. Total number of 203 up-regulated pro-inflammatory genes were detected in H5N1 group, which was larger than that of H9N2 group (141 up-regulated pro-inflammatory genes) (S9 File). In H9N2 group, macrophages generated the most pro-inflammatory factors. In H5N1 group, macrophages and M2 macrophages contributed to the most pro-inflammatory factors. These results depicted the inflammatory response landscape of various cell types from the lung after H9N2 or H5N1 AIV infection, and described the composition of immune cells and inflammatory genes during AIV-driven inflammatory response.

### The key immune cells and genes contribute to the H5N1 AIV-driven pneumonia

**Fig 5:**
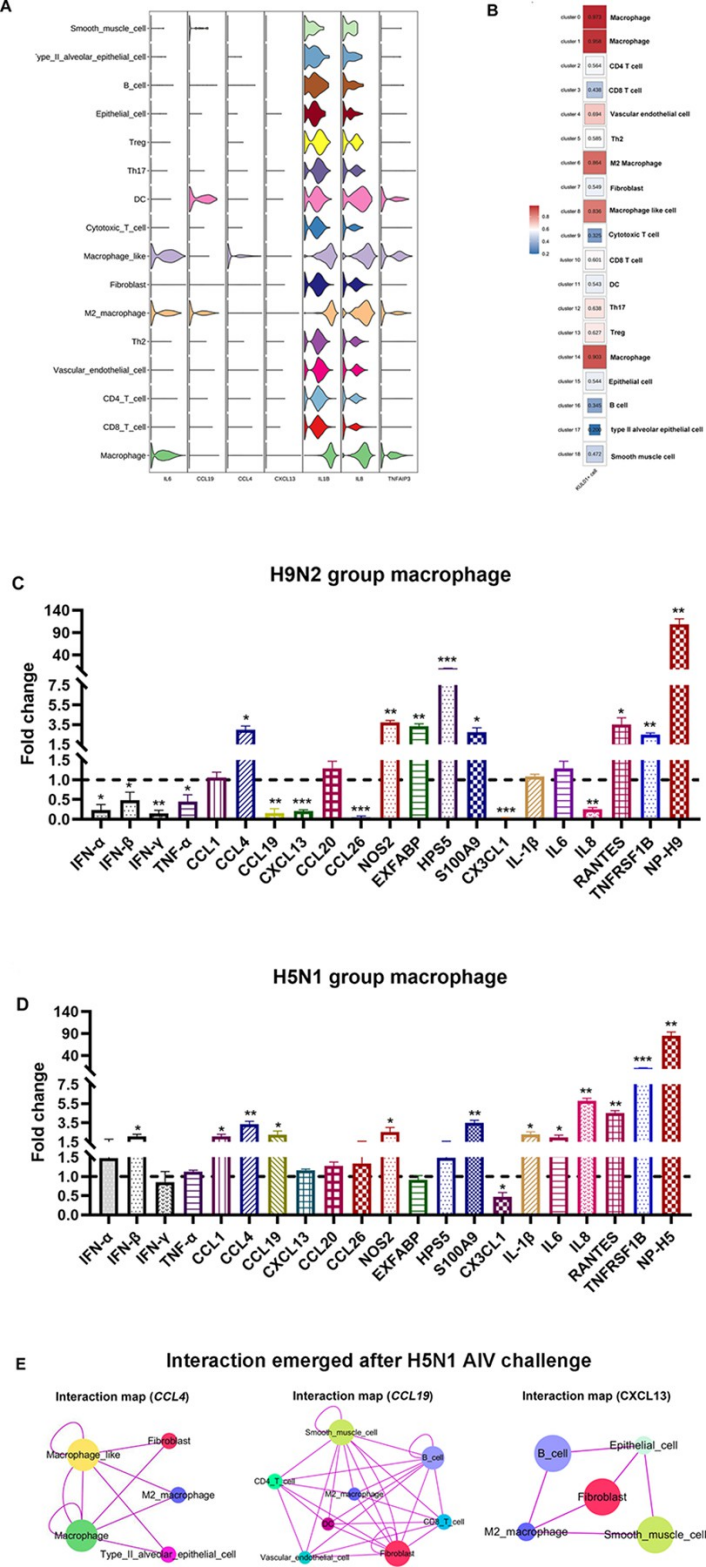
The key immune cells and genes contribute to the H5N1 AIV-driven pneumonia. (A) The normalized expression (UMI counts) of pro-inflammatory genes including IL6, CCL19, CCL4, CXCL13, IL1β, IL8, and TNFAIP3 in different cell populations. (B) Pearson’s Correlation analysis between the SMART-Seq2 data of macrophages (KUL01^+^CLASS II^+^) and lung clusters in scRNA-seq based on levels of gene expression. (C) Analysis expression of 21 differentially expressed genes (DEGs) in macrophages of single-cell lung suspensions from H9N2 AIV infected chickens at 3DPI by qRT-PCR. (D) Analysis expression of 21 DEGs in macrophages of single-cell lung suspensions from H5N1 AIV infected chickens at 1DPI by qRT-PCR. Total RNA of sorted macrophages was extracted from three chickens of the two infection groups and control groups, respectively. The data were collected from three biological samples, and each sample was tested in triplicate. The results are presented as mean ± SEM. Statistical comparisons were performed with paired t-test, and significance was assessed as P-values using GraphPad Prism. *P < 0.05, **P < 0.01, ***P < 0.001. (E) Predicted interaction map through CCL4, CCL19, and CXCL13 after H5N1 AIV challenge, respectively. Purple line indicates interactions emerging only after H5N1 AIV challenge.

To further identify the cell types involved in generating pro-inflammatory responses, we firstly analyzed the expression of pro-inflammatory factors including IL6, CCL19, CCL4, CXCL13, IL1β, IL8, and TNFAIP3 in different cell types. We confirmed that these pro-inflammatory factors were highly expressed in macrophage, M2 macrophage and macrophage like populations (Fig 5A). Besides, the Pearson’s Correlation analysis between SMART-Seq2 data of macrophages (KUL01^+^CLASS II^+^) and scRNA-seq showed that Clusters 0, 1, and 14 (annotated as a macrophage population) had high correlation with macrophages (KUL01^+^CLASS II^+^) (Fig 5B), which further increased the reliability of the annotation of scRNA-seq clusters in this study. Additionally, our foregoing results indicated that the proportion of macrophages was hugely increased in the lung from H9N2 AIV infected or H5N1 AIV infected chickens when compared to the control in scRNA-seq data (Fig 1F) and FACS validation (Fig 1H). Therefore, to explore the infiltrated macrophage induced inflammatory response, we sorted macrophages from each group and detected the expression changes of inflammatory-related genes by qRT-PCR. We found that pro-inflammatory cytokines including IFN-β, IL1β, IL6, and IL8 [51,52] and chemokines containing CCL1 and CCL19, were significantly up-regulated in macrophages of H5N1 group instead of H9N2 group (Fig 5C and 5D).

Lastly, in order to depict how cell populations interacted in the lung through pro-inflammatory cytokines and chemokines after AIV infection, we plotted the intercommunication predictions for cell types expressing the receptor or ligand of the pro-inflammatory factors. Intriguingly, we identified the interaction categories based on CCL4, CCL19, and CXCL13 and their receptors after H5N1 AIV challenge, but not H9N2 AIV challenge. Specifically, the possible communications of various cell populations in the lung through CCL4, CCL19, and CXCL13 and their receptors are sketched, which were induced after H5N1 AIV infection (Fig 5E). Thus, H5N1 AIV seemed to induce interactions of cell populations that were not present in control or H9N2 AIV infected lungs, which may contribute to the pro-inflammatory response of lung. From the above results, it can be seen that the infiltrated macrophages, pro-inflammatory cytokines including IFN-β, IL1β, IL6, and IL8, and emerging interactions of various cell populations through CCL4, CCL19, and CXCL13, may contribute to the H5N1 AIV driven inflammatory lung injury.

## Discussion

Multiple key questions related to the response of the immune cells to AIV infection in chicken lung have yet to be answered. In particular, the identity of the key immune cell types, antiviral and inflammatory factors contributing to the host protection and immunopathogenesis have not been systematically elucidated. Advances in scRNA-seq technology have allowed us to explore the atlas of the immune cells response to AIV infection in chicken lungs.

In this study, we performed 10x scRNA-seq on the sorted immune cells including MHC Class II (antigen presenting cells) and CD3 (T cells) positive cells for characterizing the responses of major immune cell populations in the lung tissue isolated from chickens after H5N1 HPAIV(1DPI), H9N2 LPAIV (3DPI) and PBS treatment. Here, 19 distinct clusters in chicken lungs were identified following the analysis of scRNA-seq data. In addition to the 11 specialized immune cell types (14 clusters) that were identified and annotated, we also identified 5 clusters of non-immune cell types which may be accidentally sorted out during the experiment. In total, 16,642 immune cells were profiled and analyzed including various T cell subsets (CD8 T cell, Cytotoxic T cell, CD4 T cell, Th2, Th17 and Treg) and antigen presentation cells (B cell, DC, Macrophage, Macrophage like cell, and M2 macrophage) (S3 and S5 Files). Moreover, we verified that the SMART-Seq2 data of sorted macrophages was highly correlated to the marker-based annotation of the macrophage population (Clusters 0, 1 and 14) (Fig 5B), which further indicated the reliability of the annotation of scRNA-seq clusters in this study. Thus for the first time (to our knowledge), we provided a valuable catalog of marker genes for identifying 16 cell types in lung tissue of chickens via scRNA-seq (Fig 1 and S3 File). In future studies, more samples and cell numbers will be necessary for identifying the complete cell atlas of the chicken lung.

Compared to LPAIV infection, the efficient virus replication of HPAIV can be correlated with tissue damage in the lung [11,12]. But the extent and nature of HPAIV and LPAIV infection in different cell types of chicken lung have not been elucidated. In this study, we found that H9N2 LPAIV seemed to be limited in infecting different cell types. Instead, H5N1 HPAIV widely infected all cell types identified by single-cell sequence (Fig 3). The difference in the tropism of cell types between LPAI and HPAI seems to align with the clinical outcome of chickens after LPAIV and HPAIV infection. In mice studies, it has been reported that influenza A Virus could infect all cell types including T cells [14,53], which is in agreement with our findings on chickens. Besides the widespread and efficient virus replication, H5N1 AIV infection induced a stronger antiviral immune response than H9N2 AIV infection in lung (Fig 2C and 2F, and S7 File). Moreover, the antiviral genes were induced and appeared in nearly all H5N1 infected cell types (Fig 2C), which indicated that the host response conferred the first line of defense in all key cell types.

HE staining results revealed that H5N1 AIV, as against H9N2 AIV infection, induced pulmonary inflammatory damage with inflammatory cell infiltration (S3 Fig), which prompted us to investigate the key immune cells and genes contributing to the H5N1 AIV-driven pneumonia. By sequentially analyzing the transcriptome of various cell types, we identified that macrophage, M2 macrophage and macrophage-like populations generated abundant pro-inflammatory factors (Figs 4 and 5A). Meanwhile, the high levels of H5N1 virus infection were discovered in these cell populations simultaneously (Fig 3E and S8 File), which reminded that massive viral replication potentially induced an excessive immune response. Besides, we found that macrophages (Clusters 0, 1 and 14) were hugely increased in the lung from H9N2 or H5N1 AIV infected chickens based on the data from scRNA-seq (Fig 1F) and FACS analysis (Fig 1G and 1H) detected using KUL01 and MHC Class II antibodies [25,54]. Compared to other cell types, macrophages demonstrated the most DEGs, especially pro-inflammatory factors responding to H9N2 and H5N1 AIV infection (Figs 2 and 4). More importantly, we found that pro-inflammatory cytokines including IFN-β, IL1β, IL6 and IL8 [51,52] and chemokines containing CCL1 and CCL19, were significantly up-regulated in the macrophages of H5N1 group as against the H9N2 group as determined by qRT-PCR (Fig 5C and 5D). Therefore, we would consider that moderate expression levels of monocytes/macrophages and inflammatory factors favor lung damage repair after H9N2 AIV infection. However, uncontrollable viral replication and cytokine release would induce serious lung immune pathology after H5N1 AIV infection. Collectively, we especially identified infiltrating macrophages with massive viral replication contributing to the excessive cytokines released and immune injury after H5N1 infection.

In mice studies, it has been reported that the communication networks between various cell types play an important role in influenza A virus (IAV)-induced cytokine storm and pneumonia [13]. Thus, we plotted the intercommunication predictions for cell types expressing the receptor or ligand of the pro-inflammatory factors. Intriguingly, we have identified the interaction categories based on the CCL4, CCL19, and CXCL13 and their receptors after H5N1 AIV challenge, as against H9N2 AIV infection (Fig 5E), which may contribute to the inflammatory lung injury. The mechanism by which the intercommunication across various cell clusters induces the release of inflammatory factors has yet to be explored.

In summary, through scRNA-seq analysis, we demonstrated the key factors associated with the pathogenesis of AIV infection in chickens. Importantly, by sequentially analyzing the transcriptome of various cell types, we discovered that infiltrating macrophages with massive viral replication and emerging interaction of various cell populations through CCL4, CCL19 and CXCL13, may contribute to the H5N1 AIV driven inflammatory lung injury. Paralleled with the previous findings from the literature, our results and validation studies confirm the fidelity of our analyses and interpretations. Our data also provide extensive resources for future studies to address the function of identified cell types and in response to pathogenic infection in chickens.

## Supporting information

S1 File Detection of the virus titer in major organs and virus shedding. (DOCX)

S2 File Primers used for qRT-PCR. (DOCX)

S3 File Cell type annotation for each cluster based on the marker genes information. (DOCX)

S4 File Marker genes expression in each cluster. (XLSX)

S5 File Details on the statistics of scRNA-seq data. (DOCX)

S6 File Differentially Expressed Genes (DEGs) in bulk or each cell type between H5N1 or H9N2-infected and uninfected groups. (XLSX)

S7 File Up-regulated DEGs involved in the GO term of “defense response to virus” (GO: 0051607) within each cell type of H5N1 group and H9N2 group. (XLSX)

S8 File Statistics of virus-infected cells and expression of viral genes within each cell types. (XLSX)

S9 File Up-regulated DEGs involved in the GO term of “inflammatory response” (GO: 0006954) within each cell type of H5N1 group and H9N2 group. (XLSX)

S1 Fig The sorting gating strategy for MHC Class II (antigen presentation cells) and CD3 (T cells) positive cells. (TIF)

S2 Fig Validation of up-regulated DEGs across all cells in the lung from H5N1 AIV (1 DPI) or H9N2 AIV (3DPI) infected chickens by qRT-PCR. Total RNA of lung cell suspensions was extracted from three chickens of the H5N1 or H9N2 AIV-infected and control groups, respectively. The data of relative mRNA expression level was derived from the ratio of the H5N1 AIV (A) or H9N2 AIV-challenge (B) group results to the control group results. qRT-PCR and RNA-seq results are respectively displayed as the 2^−ΔΔCt^ value and the average log_2_ (fold change) values of DEGs. Data from qRT-PCR were collected from three biological samples, and each sample was tested in triplicate. Statistical comparisons were performed with paired t-test, and significance was assessed as P-values using GraphPad Prism. *P < 0.05, **P < 0.01, ***P < 0.001. Error bars indicate SEM.
(TIF)

S3 Fig Hematoxylin and eosin (HE) staining and immunohistochemical (IHC) analysis of lung from the H5N1 infected, H9N2 infected and control chickens. Fresh lung tissues were firstly stained with hematoxylin and eosin, then immunohistochemically labeled for NP protein antibody, and examined microscopically. Scale bar = 100μm.
(TIF)
