# Supplementary material for: Dissection of key factors correlating with H5N1 avian influenza virus driven inflammatory lung injury of chicken identified by single-cell analysis
Source: PLoS Pathog. 2023 Oct 11;19(10):e1011685. doi: 10.1371/journal.ppat.1011685 (PMC10593216; doi:10.1371/journal.ppat.1011685)
Supplement: S1 File — (DOCX) [file ppat.1011685.s001.docx]

**Table 1. Detection of virus shedding in H5N1and H9N2 infected groups**

| Group | chicken sample number | log_10_ EID_50_/0.2 mL | |
| --- | --- | --- | --- |
|  |  | cloacal  swabs | oropharyngeal  swabs |
| Control group^a^ | #51 | ND^b^ | ND |
|  | #52 | ND | ND |
|  | #54 | ND | ND |
|  |  |  |  |
| H5N1 infected group^c^ | #66 | 1.50 | 3.75 |
|  | #67 | 1.50 | 4.25 |
|  | #68 | 2.25 | 4.50 |
|  |  |  |  |
| H9N2 infected group^d^ | #58 | 1.50 | 5.17 |
|  | #61 | 2.20 | 5.00 |
|  | #64 | 1.25 | 5.50 |

a. Control group samples were collected and detected at 0 day post infection.

b. Not detected.

c. H5N1 infected group samples were collected and detected at 1 day post infection.

d. H9N2 infected group samples were collected and detected at 3 days post infection.

**Table 2. Virus titer in different organs**

| Group | chicken  sample number | log_10_ EID_50_/0.2 mL | | |
| --- | --- | --- | --- | --- |
|  |  | Lung | Trachea | Brain |
| Control group^a^ | #51 | ND^b^ | ND | ND |
|  | #52 | ND | ND | ND |
|  | #54 | ND | ND | ND |
|  |  |  |  |  |
| H5N1 infected group^c^ | #66 | 4.75 | 5.25 | 2.25 |
|  | #67 | 5.25 | 5.25 | 2.25 |
|  | #68 | 5.50 | 4.75 | 2.50 |
|  |  |  |  |  |
| H9N2 infected group^d^ | #58 | 3.25 | 4.50 | 1.25 |
|  | #61 | 2.75 | 4.25 | 0.50 |
|  | #64 | 3.25 | 4.25 | 0.50 |

a. Control group samples were collected and detected at 0 day post infection.

b. Not detected.

c. H5N1 infected group samples were collected and detected at 1 day post infection.

d. H9N2 infected group samples were collected and detected at 3 days post infection.
