# Supplementary material for: Dissection of key factors correlating with H5N1 avian influenza virus driven inflammatory lung injury of chicken identified by single-cell analysis
Source: PLoS Pathog. 2023 Oct 11;19(10):e1011685. doi: 10.1371/journal.ppat.1011685 (PMC10593216; doi:10.1371/journal.ppat.1011685)
Supplement: S2 File — (DOCX) [file ppat.1011685.s002.docx]

**Table 3. Primers used for the qRT-PCR**

| Target | Primer Sequence (5′ - 3′) | Gen Bank No. | Reference |
| --- | --- | --- | --- |
| GAPDH | GAACATCATCCCAGCGTCCA | NM_204305.1 | (1) |
|  | CGGCAGGTCAGGTCAACAAC |  |  |
| EXFABP | CAAAGGGGTGCAGAAAATGGG | NM_205422.2 |  |
|  | TGTAGTCAGTGTCCAGCACC |  |  |
| LY6E | GTTTCTGCTCGCCGTGTTG | NM_204775.2 |  |
|  | ACTTGACAGGTGTCAGGCAG |  |  |
| OASL | ACATCCTGCCTGCTTACGAC | XM_046899821.1 |  |
|  | GTTGGGGTACTTCGGCTTCA |  |  |
| TGM4 | TGTATCCTACCTGCAGCCCT | NM_001006368.2 |  |
|  | GCACCACCTCGTTTTGAACC |  |  |
| IFIT5 | CCTGGGGGAACTACGCTTG | NM_001320422.2 |  |
|  | GGTTGTTGGGTTCTTCCCTCA |  |  |
| IFI27L1 | TCTTTCGGTGCCAAAGTCGG | NM_001002856.2 |  |
|  | GTTTCTGCGCTTCGCCATC |  |  |
| ACOD1 | CAGCTTGCGCAAAACTGCTA | NM_001030821.2 |  |
|  | TCCAACAGCCAGGGATAGGA |  |  |
| IFI6 | AGCCGGTTTCACTTCCTCTG | NM_001001296.6 |  |
|  | ATCCCACTGCTGGTAAAGCC |  |  |
| AVD | CGGGCCAGTGCTTCATAGAC | NM_205320.2 |  |
|  | TGTTGATGCCGACCCTGGTA |  |  |
| MX1 | TGCTTCACGTCAATGTCCCA | NM_204609.2 |  |
|  | TGCTCAGGCGTTTACTTGCT |  |  |
| HPS5 | ATGCAAGTGCAGACAACCCA | NM_001379276.1 |  |
|  | ATGTCCCGGTATGCTCTCCA |  |  |
| S100A9 | GCTTTGGTGAAGTGATGCTCC | NM_001305151.2 |  |
|  | GTCCTCACAGAAGTGGAGGTG |  |  |
| STAT1 | GCCCAATGGTGCATCACAAG | NM_001012914.2 |  |
|  | TGCCTACGTTTCCACTCCAC |  |  |
| IFN-α | GACAGCCAACGCCAAAGC | GU119896.1 | (1) |
|  | GTCGCTGCTGTCCAAGCATT |  |  |
| IFN-β | GCCCACACACTCCAAAACACTG | NM_001024836.1 | (1) |
|  | TTGATGCTGAGGTGAGCGTTG |  |  |
| IFN-γ | CCTCCAACACCTCTTCAACATG | X92479 | (2) |
|  | TGGCGTGCGGTCAAT |  |  |
| TNF-α | GCTGTTCTATGACCGCCCAGTT | NM_204267.1 | (1) |
|  | AACAACCAGCTATGCACCCCA |  |  |
| CCL1 | TACAGCTCCTGCTGCTACAAG | NM_204776.2 |  |
|  | CACGATAATAGCTCTGCGGGA |  |  |
| CCL4 | CTTCACCTACATCTCCCGGC | NM_001030360 | (3) |
|  | CTGTACCCAGTCGTTCTCGG |  |  |
| CCL19 | TGCCTTAGTCTCCTGGTGCT | NM_001302168.1 | (3) |
|  | CTTTGCAGTGATGAACACGGT |  |  |
| CXCL13 | GCCTGTGCCTGGTGCTC | NM_001348657.1 | (4) |
|  | TGCCCCCTTCCCCTAAC |  |  |
| CCL20 | ATGCCATCATTTTCCACACCGTCAG | NM_204438.2 | (5) |
|  | AGATGCTTCTTCACCCAGTCTTCCT |  |  |
| CCL26 | GATGGCCTACCCACAACCTG | XM_040687152.2 |  |
|  | ATGTAGGCGGAGGCAATGAG |  |  |
| NOS2 | CCACCAGGAGATGTTGAACTATGTC | NM_204961.2 |  |
|  | CCAGATGTGTGTTTTCCATGCA |  |  |
| CX3CL1 | TGCACTCAGCTCCTTGTCAG | NM_001077232.2 |  |
|  | GTAGAGCAAGGCTTCGACCA |  |  |
| IL-1β | GGTCAACATCGCCACCTACA | NM_204524.1 | (1) |
|  | CATACGAGATGGAAACCAGCAA |  |  |
| IL6 | AAATCCCTCCTCGCCAATCT | AJ309540.1 | (1) |
|  | CCCTCACGGTCTTCTCCATAAA |  |  |
| IL8 | CATCATGAAGCATTCCATCT | NM_205498.1 |  |
|  | CTTCCAAGGGATCTTCATTT |  |  |
| RANTES | CGTAGCTGTGTCCCTCTCCA | NM_001045832.2 |  |
|  | TTGTATCAGCCCCAAACGGA |  |  |
| TNFRSF1B | TTGCCACAGCTCTACCTCAC | NM_204439.4 |  |
|  | GGAACGGCCCTGTTACTGAT |  |  |
| NP-H5 | TTCCCTTCGAAAGAGCGACC |  |  |
|  | GCCCCTGGAATGACACATCT |  |  |
| NP-H9 | TCCCCTTTGAAAGAGCGACC |  |  |
|  | GGTTCGTTGCCTTTTCGTCC |  |  |

**References:**

1. Dai M, Wu S, Feng M, Feng S, Sun C, Bai D, et al. Recombinant chicken interferon-alpha inhibits the replication of exogenous avian leukosis virus (ALV) in DF-1 cells. MOL IMMUNOL. 2016 2016-08-01; 76:62-9.

2. Liu D, Qiu Q, Zhang X, Dai M, Qin J, Hao J, et al. Infection of chicken bone marrow mononuclear cells with subgroup J avian leukosis virus inhibits dendritic cell differentiation and alters cytokine expression. INFECT GENET EVOL. 2016 2016-10-01; 44:130-6.

3. Hong Y, Lee J, Vu TH, Lee S, Lillehoj HS, Hong YH. Exosomes of lipopolysaccharide-stimulated chicken macrophages modulate immune response through the MyD88/NF-kappaB signaling pathway. DEV COMP IMMUNOL. 2021 2021-02-01; 115:103908.

4. Hong Y, Lee J, Vu TH, Lee S, Lillehoj HS, Hong YH. Chicken avian beta-defensin 8 modulates immune response via the mitogen-activated protein kinase signaling pathways in a chicken macrophage cell line. Poult Sci. 2020 2020-09-01;99(9):4174-82.

5. Luo C, Liu J, Qi W, Ren X, Lu R, Liao M, et al. Dynamic analysis of expression of chemokine and cytokine gene responses to H5N1 and H9N2 avian influenza viruses in DF-1 cells. MICROBIOL IMMUNOL. 2018 2018-05-01;62(5):327-40.
