# Supplementary material for: Dissection of key factors correlating with H5N1 avian influenza virus driven inflammatory lung injury of chicken identified by single-cell analysis
Source: PLoS Pathog. 2023 Oct 11;19(10):e1011685. doi: 10.1371/journal.ppat.1011685 (PMC10593216; doi:10.1371/journal.ppat.1011685)
Supplement: S3 File — (DOCX) [file ppat.1011685.s003.docx]

| **Cluster** | **Signature genes** | **Cell type annotation** |
| --- | --- | --- |
| Cluster 3/10 | CD3D, CD8A, IL2RB, GNLY, GranzymeK, GranzymeA | CD8^+^ T cell |
| Cluster 9 | CD3D, CD8A, IL2RB, GNLY, GranzymeK, GranzymeA, IFNG | Cytotoxic T cell |
| Cluster 2 | CD3D, SATB1, CD28, IL7R, CD4 | CD4^+^ T cell |
| Cluster 5 | CD3D, DRD4, KK34, MAF, CCR4 | Th2 cell |
| Cluster 12 | CD3D, CCR6, IL17A, ICOS, RORA, CCL20 | Th17 cell |
| Cluster 13 | CD3D, CD25, IL7R, TNFRSF1B | Treg cell |
| Cluster 16 | BLB2, Bu-1, BCL11A, CD79B | B cell |
| Cluster 11 | BLB2, XCR1, SNX22, CD80, CD86, CD83 | DC |
| Cluster 0/1/14 | BLB2, CSF3, NOS2, IL1B, VCAN, RNASE6 | Macrophage |
| Cluster 8 | BLB2, HSPH1, DNAJA4, IL1B, VCAN | Macrophage like |
| Cluster 6 | BLB2, RNASE6, VSIG4, IL1B | M2 macrophage |
| Cluster 4 | ADGRL4, VWF, PODXL, SelectinE | Vascular endothelial cell |
| Cluster 7 | COL1A1, COL1A2, COL6A1 | Fibroblasts |
| Cluster 15 | BMX, EHF | Epithelial cell |
| Cluster 17 | SFTPA1, SFTPA2, SFTPC | type II alveolar epithelial cell |
| Cluster 18 | MYH11, TAGLN | Smooth muscle cell |

**Table 4. Cell type annotation based on the marker genes information**
