# Supplementary material for: Dissection of key factors correlating with H5N1 avian influenza virus driven inflammatory lung injury of chicken identified by single-cell analysis
Source: PLoS Pathog. 2023 Oct 11;19(10):e1011685. doi: 10.1371/journal.ppat.1011685 (PMC10593216; doi:10.1371/journal.ppat.1011685)
Supplement: S5 File — (DOCX) [file ppat.1011685.s005.docx]

**Table 5. scRNA-seq data statistics**

| **Sample** | **Number of Reads** | **Valid Barcodes** | **Number of Cells (before_filter)** | **Number of Cells (after_filter)** | **Median UMI Counts per Cell** | **Median genes per Cell** | **Mapped to Genome** |
| --- | --- | --- | --- | --- | --- | --- | --- |
| **Control** | 404,691,253 | 95.8% | 14240 | 5736 | 988 | 557 | 77.6% |
| **H5N1-group** | 401,418,283 | 95.3% | 11178 | 6779 | 1923 | 826 | 78.1% |
| **H9N2-group** | 554,037,694 | 95.5% | 16811 | 6936 | 1117 | 606 | 72.1% |

**Table 6.** **Number of cells in each cluster and their proportional distribution in each group**

| **Cluster** | **Control** | **H5N1-group** | **H9N2-group** |
| --- | --- | --- | --- |
| **Total** | 5736 (100%) | 6779 (100%) | 6936 (100%) |
| **0** | 147 (2.56%) | 1655 (24.41%) | 999 (14.4%) |
| **1** | 348 (6.07%) | 1302 (19.21%) | 1028 (14.82%) |
| **2** | 1658 (28.91%) | 142 (2.09%) | 668 (9.63%) |
| **3** | 814 (14.19%) | 611 (9.01%) | 810 (11.68%) |
| **4** | 475 (8.28%) | 135 (1.99%) | 1056 (15.22%) |
| **5** | 664 (11.58%) | 153 (2.26%) | 736 (10.61%) |
| **6** | 531 (9.26%) | 665 (9.81%) | 325 (4.69%) |
| **7** | 354 (6.17%) | 238 (3.51%) | 360 (5.19%) |
| **8** | 27 (0.47%) | 798 (11.77%) | 44 (0.63%) |
| **9** | 176 (3.07%) | 305 (4.5%) | 368 (5.31%) |
| **10** | 99 (1.73%) | 231 (3.41%) | 164 (2.36%) |
| **11** | 141 (2.46%) | 224 (3.3%) | 50 (0.72%) |
| **12** | 111 (1.94%) | 118 (1.74%) | 151 (2.18%) |
| **13** | 67 (1.17%) | 61 (0.9%) | 45 (0.65%) |
| **14** | 9 (0.16%) | 53 (0.78%) | 82 (1.18%) |
| **15** | 88 (1.53%) | 5 (0.07%) | 15 (0.22%) |
| **16** | 14 (0.24%) | 32 (0.47%) | 16 (0.23%) |
| **17** | 11 (0.19%) | 21 (0.31%) | 15 (0.22%) |
| **18** | 2 (0.03%) | 30 (0.44%) | 4 (0.06%) |
