# Supplementary figures and images for: Dissection of key factors correlating with H5N1 avian influenza virus driven inflammatory lung injury of chicken identified by single-cell analysis
Source: PLoS Pathog. 2023 Oct 11;19(10):e1011685. doi: 10.1371/journal.ppat.1011685 (PMC10593216; doi:10.1371/journal.ppat.1011685)

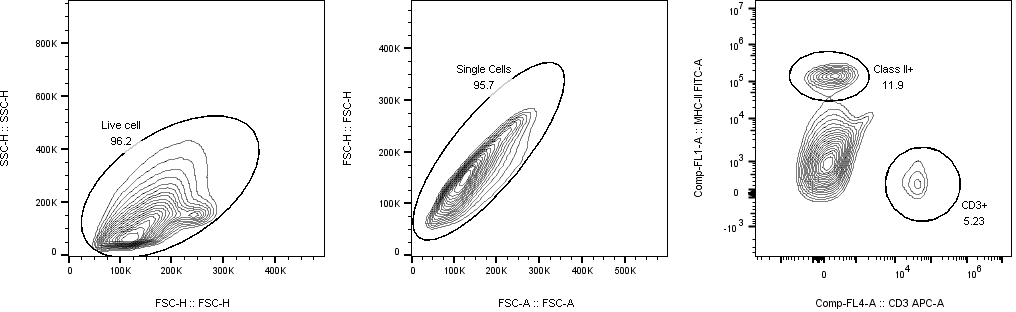

Supplement: S1 Fig — (TIF) [file ppat.1011685.s010.tif]

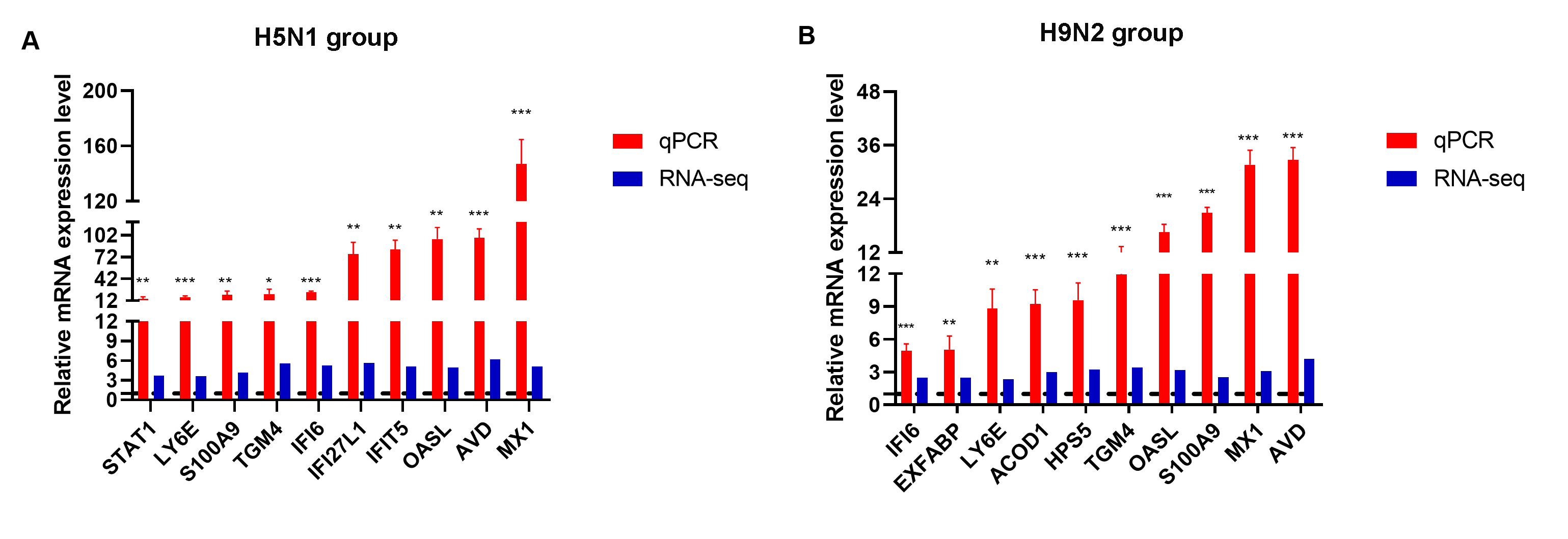

Supplement: S2 Fig — Total RNA of lung cell suspensions was extracted from three chickens of the H5N1 or H9N2 AIV-infected and control groups, respectively. The data of relative mRNA expression level was derived from the ratio of the H5N1 AIV (A) or H9N2 AIV-challenge (B) group results to the control group results. qRT-PCR and RNA-seq results are respectively displayed as the 2−ΔΔCt value and the average log2 (fold change) values of DEGs. Data from qRT-PCR were collected from three biological samples, and each sample was tested in triplicate. Statistical comparisons were performed with paired t-test, and significance was assessed as P-values using GraphPad Prism. *P < 0.05, **P < 0.01, ***P < 0.001. Error bars indicate SEM. (TIF) [file ppat.1011685.s011.tif]

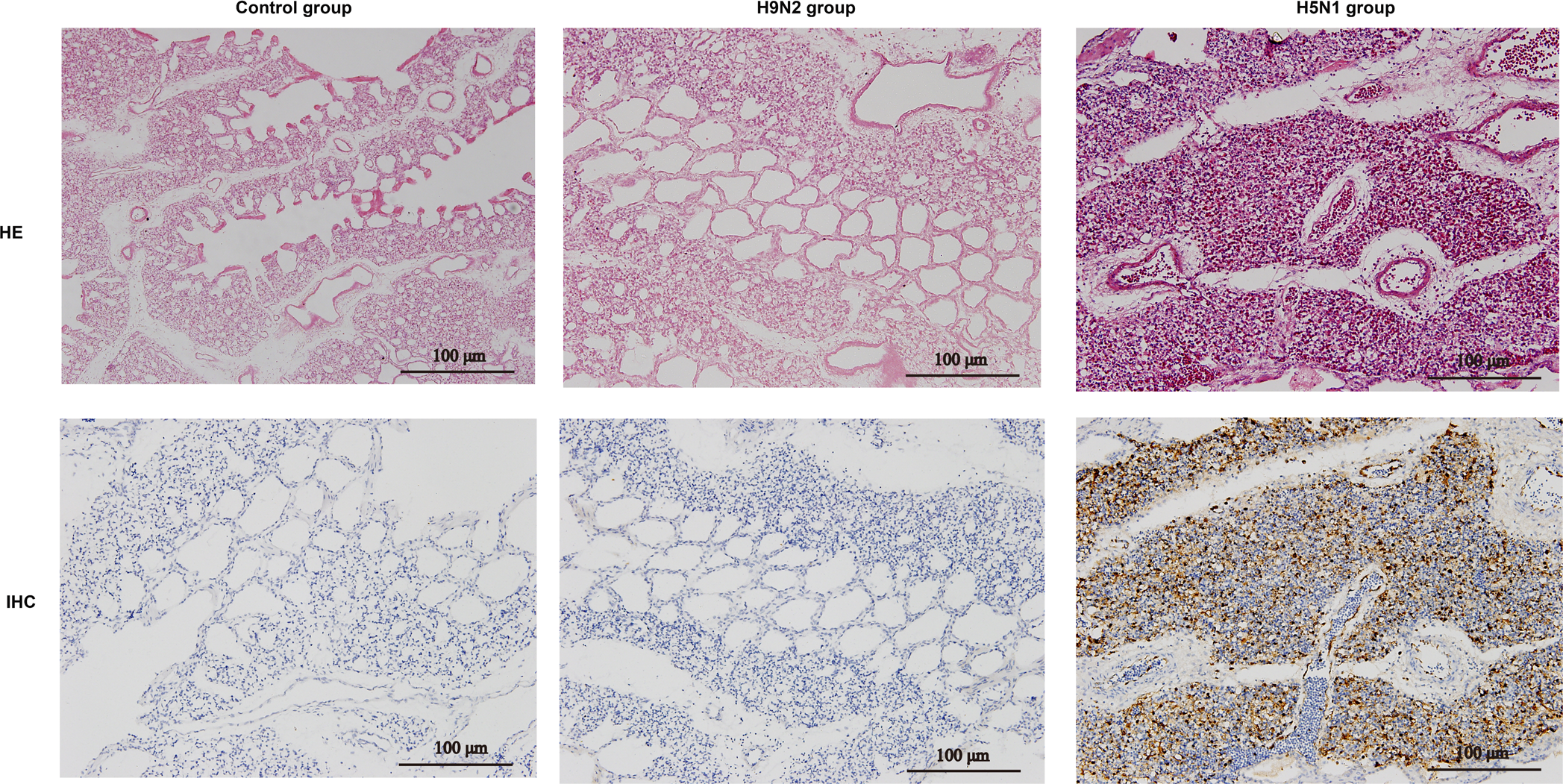

Supplement: S3 Fig — Fresh lung tissues were firstly stained with hematoxylin and eosin, then immunohistochemically labeled for NP protein antibody, and examined microscopically. Scale bar = 100μm. (TIF) [file ppat.1011685.s012.tif]
